# The Import of the Sweating of Consumptives

**Published:** 1881-05

**Authors:** 


### THE IMPORT OF THE SWEATING OF CONSUMPTIVES.

Dr. Rousselot, of Saint-Die, discusses in the *Revue Med. de l' Est* some of the peculiarities of phthisical sweating, the variable period of the appearance of this symptom, and the point whether in phthisical persons it is to be considered an evil symptom, and one which is to be combatted. M. Rousselot believes that, in a certain number of cases, there is a correlation between the sweating and the fever. He remarks, in the first instance, that nothing is more variable than the period of appearance of the sweating in the course of pulmonary phthisis. There is an active tubercular evolution and a torpid evolution in some sort passive. In the second case, pulmonary lesion has no influence on the organism. It has not an effective evolution, and it may last for some time without producing fever, and, in consequence, without the procession of symptoms which are ordinarily observed with fever, and particularly in nocturnal sweating. When, on the contrary, there are, from the onset, an active evolution, nocturnal fever, and disordered condition of all these symptoms, then, in general, a hasty appearance of nocturnal sweating may be observed. In this case, the thermometer will render great service in enabling us to study the degrees of morbid combustion. The sweating, which is then very often extremely abundant, allows the elimination of a great quantity of the products of morbid combustion. It may, then, be admitted, according to M. Roueselot, that the sweating affords a derivation favorable to the fever, and does, to some extent, moderate that symptom. If then, in certain tubercular persons, nocturnal sweating appears, as it were, at the outset of the affection, it is because these individuals have a tuberculous evolution of an active form, and one which tends to fluxion and precocious fever. In others, on the contrary, the evolution effects a silent, indolent, torpid form, without any recoil on the organism; or more strictly, there are tubercles in the lung, but no tuberculous evolution, and the subject is not phthisical.—*N. Y. Medical Gazette.*

